# Application of the transformer model algorithm in chinese word sense disambiguation: a case study in chinese language

**DOI:** 10.1038/s41598-024-56976-5

**Published:** 2024-03-15

**Authors:** Linlin Li, Juxing Li, Hongli Wang, Jianing Nie

**Affiliations:** 1 The College of Literature and Journalism, Sichuan University, Chengdu, 610000 China; 2 School of Journalism and New Media, Xi’an Jiaotong University, Xi’an, 710049 China; 3 School of Artificial Intelligence, Tiangong University, Tianjin, 300000 China; 4 School of Art, College of International Business and Economics, Wuhan Textile University, Wuhan, 430000 China

**Keywords:** Transformer model algorithm, Chinese language, BiLSTM, Word sense disambiguation, Root mean squared error, Computer science, Information technology

## Abstract

This study aims to explore the research methodology of applying the Transformer model algorithm to Chinese word sense disambiguation, seeking to resolve word sense ambiguity in the Chinese language. The study introduces deep learning and designs a Chinese word sense disambiguation model based on the fusion of the Transformer with the Bi-directional Long Short-Term Memory (BiLSTM) algorithm. By utilizing the self-attention mechanism of Transformer and the sequence modeling capability of BiLSTM, this model efficiently captures semantic information and context relationships in Chinese sentences, leading to accurate word sense disambiguation. The model’s evaluation is conducted using the PKU Paraphrase Bank, a Chinese text paraphrase dataset. The results demonstrate that the model achieves a precision rate of 83.71% in Chinese word sense disambiguation, significantly outperforming the Long Short-Term Memory algorithm. Additionally, the root mean squared error of this algorithm is less than 17, with a loss function value remaining around 0.14. Thus, this study validates that the constructed Transformer-fused BiLSTM-based Chinese word sense disambiguation model algorithm exhibits both high accuracy and robustness in identifying word senses in the Chinese language. The findings of this study provide valuable insights for advancing the intelligent development of word senses in Chinese language applications.

## Introduction

Natural Language Processing (NLP), a vital branch of artificial intelligence, aims to facilitate computers in understanding, processing, and generating human language. Among the diverse tasks within NLP, word sense disambiguation is a challenging and fundamental problem^1–3^. In complex languages like Chinese, word sense ambiguity is more pronounced due to the polysemy of words and the intricacies of context. For instance, a word may carry different meanings based on context, presenting significant challenges in text comprehension and semantic reasoning^4,5^. Consequently, it becomes essential to address word sense ambiguity in the Chinese language and achieve accurate word sense disambiguation to enhance the effectiveness of NLP applications.

Traditional approaches in Chinese word sense disambiguation encounter certain limitations. While word vector representation methods like Word2Vec can capture semantic information to some extent, they struggle with handling long-range dependencies and complex sentence structures^6^. Conversely, conventional sequence models such as Long Short-Term Memory (LSTM) can consider contextual information. Still, they may face challenges like vanishing or exploding gradients when dealing with lengthy sequences, leading to limited effectiveness^7^. The Transformer model presents an innovative sequence modeling algorithm with a self-attention mechanism to overcome these limitations. This mechanism efficiently captures correlations between different positions in the input sequence, enabling parallel computation and capturing long-range dependencies^8,9^. Furthermore, the integration of Bi-directional Long Short-Term Memory (BiLSTM), which considers both forward and backward contexts, enhances the model’s ability to comprehend sentence semantics^10,11^.

In conclusion, this study presents a novel application of the Transformer model algorithm to address Chinese word sense ambiguity through word sense disambiguation. By combining the self-attention mechanism of Transformer with the sequence modeling capability of BiLSTM, an efficient and accurate Chinese word sense recognition model is constructed.

The significance of this study can be summarized as follows: Firstly, it explores a practical approach to applying the Transformer model to Chinese word sense disambiguation tasks, contributing new perspectives to Chinese NLP. This study highlights the integration of the self-attention mechanism from the Transformer model and the sequence modeling capability of the BiLSTM algorithm. By combining these elements, the model excels in capturing semantic information and contextual relationships within Chinese sentences, leading to enhanced accuracy in word sense disambiguation. This improved accuracy is substantiated by experimental results from the PKU Paraphrase Bank, where the model achieved an accuracy of 83.71%, surpassing the performance of the LSTM algorithm. Secondly, it enhances the accuracy and robustness of Chinese word sense disambiguation, providing practical solutions to the challenge of word sense ambiguity. Lastly, this study lays the foundation for advancing Chinese semantic intelligence and offers substantial support for applications in intelligent dialogue, machine translation, text generation, and related fields. As a result, the findings of this study hold promise for future research and development in the field of Chinese language processing and semantic understanding. The innovation in this study manifests across multiple dimensions. Firstly, the research explores the application of the Transformer model in Chinese word sense disambiguation tasks, introducing novel ideas and methodologies to the realm of Chinese NLP. By integrating the Transformer model with the BiLSTM algorithm, the study comprehensively addresses the intricacies of the Chinese context in model construction, effectively tackling the challenge of Chinese word sense ambiguity. Linguistic disparities in structures, expressions, and grammar rules between Chinese and English directly impact approaches to word sense disambiguation tasks. To accommodate these distinctions, the study adapts the Transformer and BiLSTM to align more closely with the linguistic characteristics of Chinese. Through thorough adjustments and optimizations, the model ensures its capacity to accurately capture semantic information and contextual relationships in the Chinese language, thereby enhancing word sense disambiguation performance. Furthermore, by leveraging the self-attention mechanism of the Transformer and the sequence modeling capability of BiLSTM, the designed model adeptly captures semantic information and contextual relationships in Chinese sentences. This integrated design enhances the model’s accuracy in Chinese word sense disambiguation, offering a fresh perspective for addressing natural language understanding challenges in Chinese contexts. The study meticulously selects a Chinese dataset (PKU Paraphrase Bank) that closely aligns with the Chinese context for model evaluation, avoiding merely adapting English datasets to the Chinese environment. This careful dataset selection, coupled with profound model optimizations for the Chinese context, underscores the innovation and distinctiveness of this study in addressing the intricacies of Chinese word sense disambiguation. The synergistic combination of dataset selection and innovative model structure design ensures the adaptability and high performance of the model for Chinese word sense disambiguation tasks. The study explores various facets, including methodology, model design, and dataset selection, presenting a novel approach for Chinese word sense disambiguation tasks. Experimental results showcase the model’s high accuracy and robustness in the Chinese context, contributing new insights to the field of Chinese NLP and practical solutions for future research and applications in domains such as intelligent dialogue, machine translation, and text generation.

## Literature review

### Research status of Chinese word sense disambiguation and word sense ambiguity

As a complex and linguistically diverse language, Chinese has been a prominent research focus in the NLP field concerning word sense disambiguation and ambiguity. Word sense disambiguation involves comprehending the specific meaning of a word within varying contextual environments. Conversely, word sense ambiguity pertains to a word having multiple meanings that necessitate precise identification based on the context^12^. Numerous scholars have made significant contributions to this area of research. Li et al^13^. revealed the existence of universal and specific reading mechanisms under different writing systems. Their findings shed light on cross-linguistic differences in the reading process, offering valuable insights into the universality and specificity of the reading system. In the work of Wang et al^14^., a robust pre-training language model was proposed for oral comprehension, exhibiting efficient performance on verbal comprehension tasks. Søgaard^15^ explored methods to establish word vector spaces from raw text to enhance the semantic meaning of words, demonstrating its effectiveness in handling semantic information. Chen and Chen^16^ uncovered that long-term semantic representations of known vocabulary undergo updates and integration with new information during second language learning, revealing the dynamic nature of semantic learning. Collectively, these studies contribute to the advancement of word sense understanding and disambiguation in the context of the Chinese language.

### Transformer model and its current applications in NLP

The Transformer model, a deep learning architecture built upon attention mechanisms, has gained considerable prominence due to its robust modeling capabilities and advantages in parallel computing. Its performance has been remarkable across various NLP tasks, including language modeling, machine translation, and text classification. Numerous researchers in relevant fields have conducted in-depth investigations on this model. Von der Mosel et al^17^. explored the efficacy of pre-trained Transformers in NLP for software engineering. The research substantiated the practicality of employing pre-trained Transformers in this context, providing valuable empirical evidence. Peer et al^18^. proposed a greedy layer pruning method to accelerate Transformer models in NLP. This approach effectively compressed the model size, improving training and inference speeds. Yang et al^19^. compared Transformers and traditional NLP methods in radiology report classification. The findings revealed that Transformer models necessitated fewer data to achieve automated report classification than conventional methods. Moreover, Remedios and Remedios^20^. explored the potential of applying the Transformer model to large-scale language processing in clinical radiology, demonstrating its positive impact on advancing NLP applications in this domain.

### BiLSTM model and its advantages in sequence modeling

The BiLSTM network stands as a widely employed model in the field of sequence modeling. This model exhibits the unique capability to consider both past and future information of the input sequence simultaneously, enabling it to effectively capture dependencies between contexts. Consequently, BiLSTM excels in tasks that demand word sense recognition. Extensive research has been conducted on the application of BiLSTM by various scholars. Xu^21^ explored the use of BiLSTM in intelligent human–computer interaction (HCI) within the domain of deep learning. The findings underscored the potential of deep learning in achieving intelligent HCI. Sornlertlamvanich & Yuenyong^22^ utilized the Bidirectional Long Short-Term Memory—Convolutional Neural Network—Conditional Random Field model in conjunction with the Thai character cluster method to implement Thai-named entity recognition. The results demonstrated the effectiveness of this approach in the Thai-named entity recognition task. Ma et al^23^. proposed a graph-enhanced sequence-to-sequence model for neural question-answering generation, revealing its strong performance in the question-answering generation task. Additionally, Ye et al^24^. employed the BiLSTM algorithm and Support Vector Machine Naive Bayes classifier for text sentiment recognition, with the method achieving commendable performance in sentiment recognition.

## Summary

In the field of Chinese lexical semantics disambiguation, numerous researchers and scholars have delved deeply into exploration and study. These methods can generally be categorized into two types: rule-based methods and machine learning-based methods. Rule-based methods typically rely on the expertise of linguists and a profound understanding of the characteristics of the Chinese language. These methods achieve the disambiguation of meanings by formulating a series of rules, such as rules for selecting meanings and context-matching rules. However, a major drawback of this approach is its difficulty in addressing complex language phenomena and the ever-changing nature of meanings. Additionally, rule design often depends on the personal experience and subjective judgment of linguists, lacking objectivity and universality. Machine learning-based methods, on the other hand, usually leverage large-scale corpora for training and automatically learn rules and patterns for lexical semantics disambiguation. Traditional machine learning algorithms such as support vector machines naive Bayes, as well as deep learning algorithms like convolutional neural networks, recurrent neural networks, etc., are widely applied to disambiguation tasks. However, the performance of these models is often influenced by the quality and quantity of the training data. If the training data is of low quality or insufficient, the model’s performance may be constrained.

Through the analysis of the research mentioned above, it is evident that, previous research on Chinese word sense disambiguation and ambiguity has been characterized by its scattered nature, lacking systematic integration and in-depth exploration.

Although scholars have recognized differences in language structure and grammar rules, there has been relatively little exploration of the application of deep learning models to address Chinese word sense ambiguity tasks. Secondly, the current Transformer model has demonstrated outstanding performance in the field of NLP. However, previous research has primarily concentrated on its application in specific domains (such as software engineering and clinical radiology), with a paucity of systematic studies on the Transformer in Chinese word sense disambiguation tasks. This underscores the need for a comprehensive examination to validate the applicability and performance of the Transformer model in the Chinese context. Additionally, while BiLSTM has achieved significant success in the field of sequence modeling, previous research has predominantly centered on its application in various tasks, with limited integration with models like the Transformer, especially in the context of Chinese word sense disambiguation tasks. This motivates the endeavor to integrate Transformer and BiLSTM to leverage their respective strengths, with the aim of enhancing the accuracy of Chinese word sense disambiguation. Meanwhile, the application of deep learning models in various fields is common. For instance, BiLSTM is widely used in sequence modeling across different domains, while a Transformer is commonly employed in NLP tasks such as language modeling, machine translation, and text classification. However, applying Transformer to Chinese word sense recognition still presents significant challenges. Therefore, this study aims to fuse BiLSTM and Transformer to be used in Chinese word sense recognition, intending to contribute valuable insights and guidance for the accurate processing and credit of the Chinese language in future endeavors.

## Chinese word sense recognition method based on Transformer and BiLSTM

### Chinese word sense dataset and preprocessing analysis

In the field of NLP, Chinese word sense recognition plays a crucial role in multiple tasks and applications. Chinese word sense recognition refers to determining the specific meaning or semantics of a word in a given context. Due to the polysemy or multiple pronunciations of many Chinese words, a single word may have several distinct meanings^25,26^. Therefore, Chinese word sense recognition is essential for accurately understanding the semantic content of sentences or texts. Table 1 illustrates the polysemy of the same Chinese word in different contexts.

**Table 1:** List of examples of polysemy in Chinese.

	Word	Meaning 1	Meaning 2
Different meaning	买	Purchase items	Win a competition
	学	Acquire knowledge	Go to school
	红	Color: Red	State of affairs: loss, failure
	头	Body part: Head	One end of an object: Pen tip, knife edge
Different sound	重	zhòng (weight)	chóng (repeat)
	行	xíng (to walk)	háng (bank, company)
	打	dǎ (to make a phone call)	dá (quantifier, a dozen eggs)
	了	le (already)	liǎo (to understand)

Therefore, in tasks such as text analysis, information retrieval, and machine translation, word sense disambiguation plays a crucial role in ensuring the correct comprehension and expression of sentence meanings. In information retrieval, search engines rely on accurately identifying word senses to return relevant search results. In machine translation, precise recognition of word senses in the source language sentence aids the translation system in selecting more accurate translation results. Additionally, Chinese word sense disambiguation is crucial in accurately understanding the overall meaning and sentiment orientation of entire texts. Consequently, it is essential for tasks such as text comprehension, sentiment analysis, and question-answering systems.In this study, the task of word sense ambiguity is defined as addressing the challenge unique to the Chinese context, where a vocabulary item may possess multiple meanings (senses). The study specifically aims to develop an effective method capable of accurately identifying and distinguishing the specific meanings of the same word in different contexts. This task is grounded in the recognition that Chinese, as a language, exhibits rich grammatical structures and complex semantic expressions, resulting in the possibility that the same word may carry multiple meanings in different contexts. For example, many Chinese vocabulary items may share similar spelling or pronunciation in different contexts but convey significantly different meanings. Consequently, resolving the issue of word sense ambiguity is paramount for enhancing the accuracy and deep understanding of NLP tasks in Chinese. In the Chinese context, word sense ambiguity encounters a series of unique challenges. First, the expressive manner of Chinese is relatively flexible, and a word may convey multiple meanings through different combinations. Second, the common occurrence of homophones in Chinese increases the difficulty of word sense ambiguity, as a word with the same pronunciation may have entirely different meanings in different contexts. Additionally, the grammatical structure and expression style of Chinese differs significantly from languages like English, necessitating in-depth research on how to effectively capture semantic information in Chinese sentences to resolve ambiguity.

**Figure 1:**
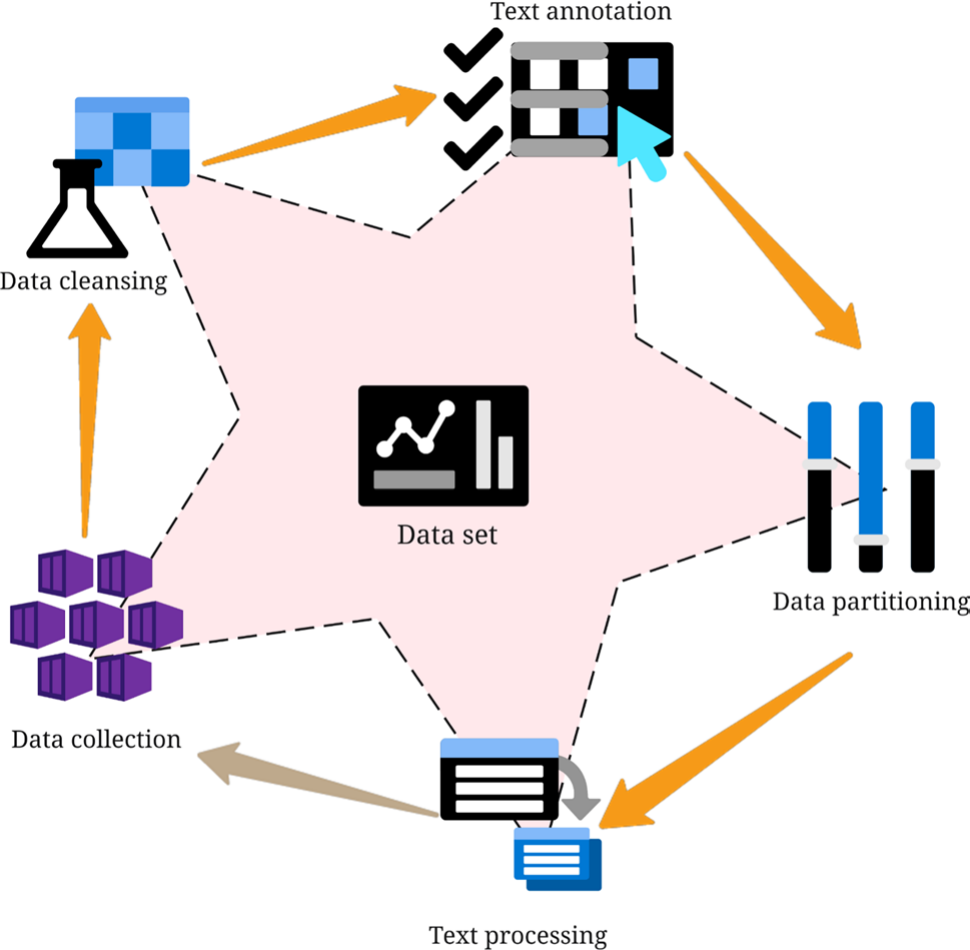
Data preprocessing flowchart.

This study selected the PKU-Paraphrase-Bank (https://github.com/pkucoli/PKU-Paraphrase-Bank/), a Chinese text paraphrase dataset, as the dataset^27^. Peking University developed the dataset to assist researchers in conducting studies related to text paraphrasing, such as text similarity, sentence generation, and semantic understanding. Each dataset entry consists of two columns representing two sentences with the same meaning, separated by “\t”. It contains a total of 509,832 pairs of sentences, with an average of 23.05 words per sentence^28^. The specific preprocessing flow for applying this dataset to Chinese word sense disambiguation is illustrated in Fig. 1.

As depicted in Fig. 1, the preprocessing of the dataset begins with data selection and collection, involving many Chinese sentence pairs. These sentence pairs consist of paraphrased sentences with similar meanings. The subsequent step involves data cleaning to eliminate possible noise, irrelevant or redundant information, and duplicate data, thereby ensuring data quality and accuracy. Following data cleaning, text annotation is performed, wherein each text pair is labeled with a binary label indicating the relationship between the two sentences. Label 1 signifies that the two sentences are similar (paraphrase relationship), while label 0 demonstrates that the two sentences are dissimilar (non-paraphrase relationship). Subsequently, the data is split into training and testing sets in an 8:2 ratio. Lastly, text processing is executed, encompassing tokenization, stop word removal, and stemming, among other steps, to furnish cleaner and more concise inputs for the model.

### Transformer model and attention mechanism analysis

**Figure 2:**
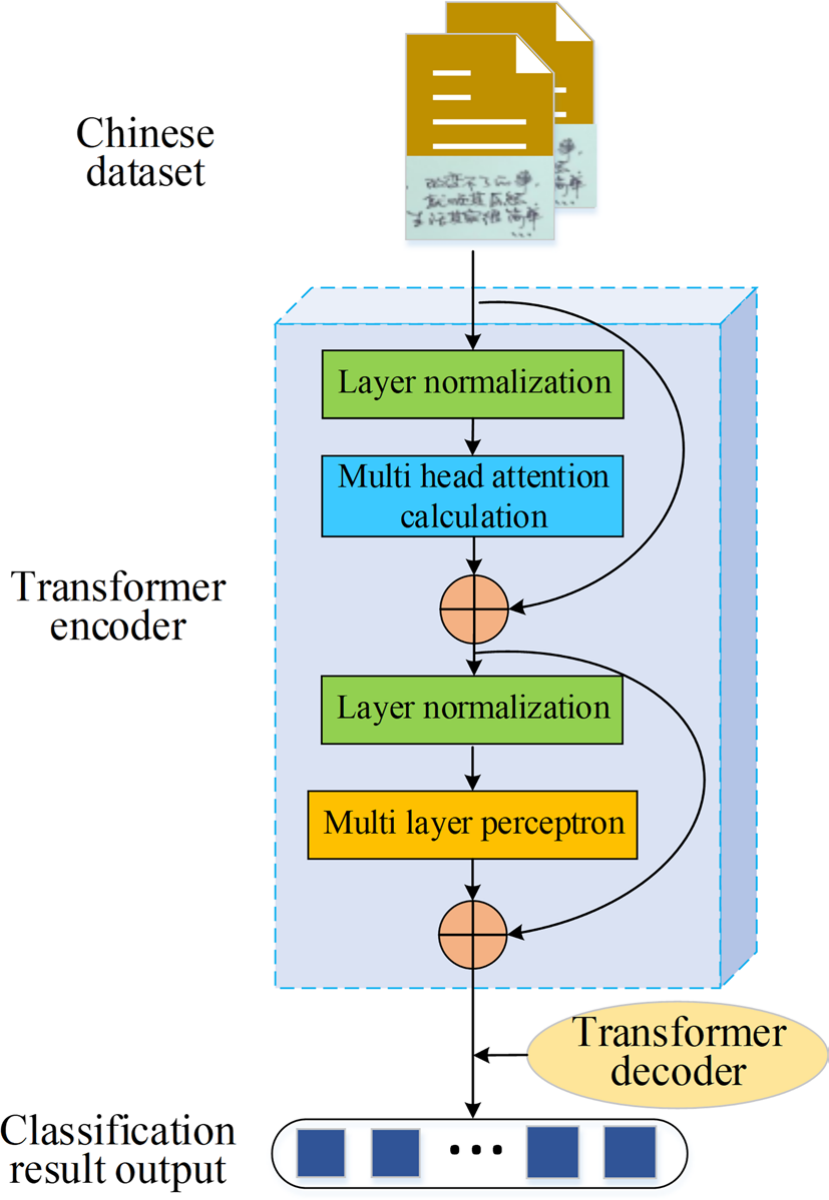
Application of the Transformer model framework in NLP.

The Transformer model consists of an encoder and a decoder. The encoder processes input text, while the decoder generates the output text. The model finds extensive application in tasks such as machine translation^29,30^. Figure 2 illustrates the application of the Transformer model framework in NLP.

As depicted in Fig. 2, the Transformer model processes the corpus into word vectors, which are then input to the encoder. Each word vector *x*_*i*_ in the matrix *X* undergoes multiplication with three weight matrices, yielding the query vector (*Q*), key vector (*K*), and value vector (*V*). In the self-attention layer, attention is computed between each word of the Chinese ambiguous term and the sentence. Equation (1) describes the calculation for attention, represented as *Attention*(*Q*, *K*, *V*).

1 $$Attention(Q,K,V) = soft\max \left( {\frac{{Q \cdot K^{T} }}{{\sqrt {d_{k} } }}} \right) \cdot V$$

where *Q* represents the matrix of “query” vectors, *K* denotes the matrix of “key” vectors, and *V* signifies the matrix of “value” vectors. The process of Chinese word sense disambiguation using the Transformer model involves data preprocessing, disambiguation feature extraction, and semantic classification. The pseudocode for this specific process is illustrated in Algorithm 1.

**Algorithm 1:**
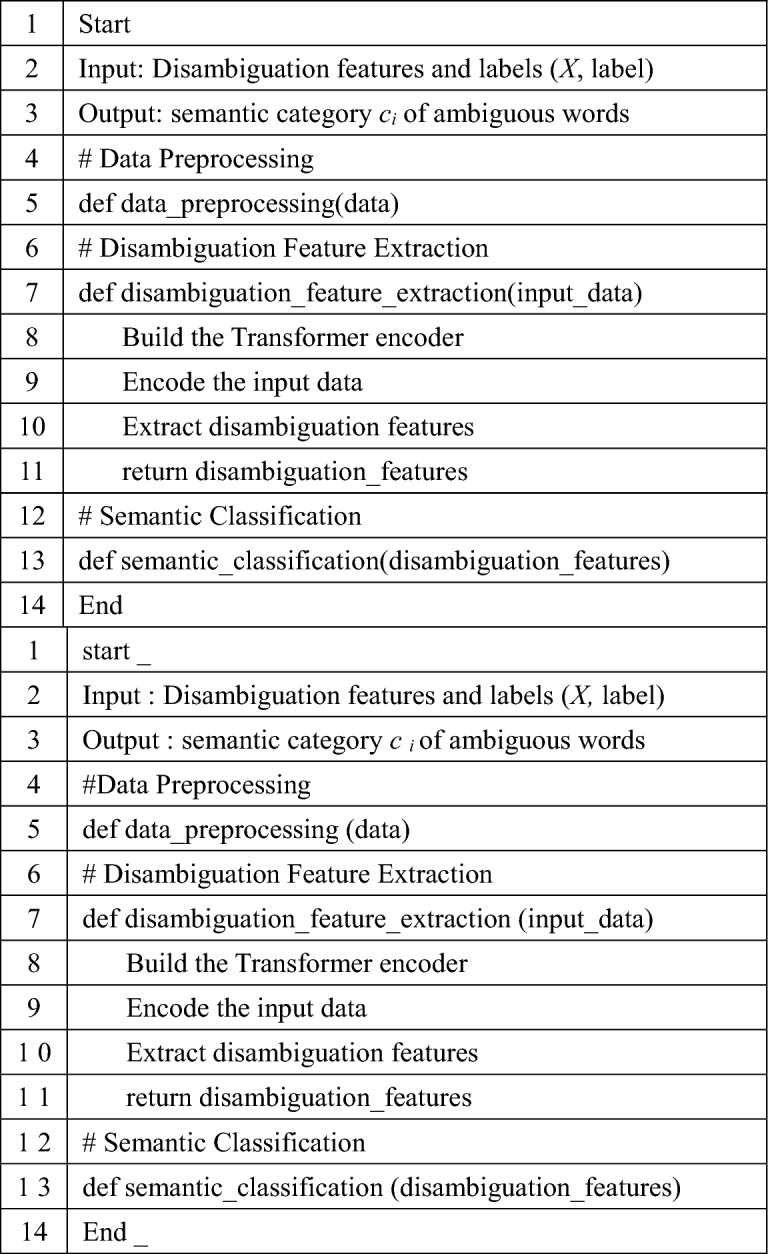
**Pseudocode for applying the Transformer model to Chinese word sense disambiguation**

In this process, the experiment leverages the multi-head attention mechanism to extract disambiguation features from multiple perspectives^31^. The variation of the multi-head attention mechanism includes N linear transformations on matrices *Q*, *K*, and *V*, and its calculation is as follows:

2 $$MultiHeadAtt = concat\left( {Att_{1} ,Att_{2} , \cdots ,Att_{N} } \right) \cdot W^{O}$$

where $W^{O}$ refers to the linear mapping matrix that fuses the *N* representations. The formula for $Att_{i}$ is given by Eq. (3).

3 $$Att_{i} = \frac{{\left( {\frac{{Q_{i} \cdot K_{{_{i} }}^{T} }}{{\sqrt {d_{k} } }}} \right)}}{{\sum\limits_{i} {\left( {\frac{{Q_{i} \cdot K_{{_{i} }}^{T} }}{{\sqrt {d_{k} } }}} \right)} }} \cdot V_{i}$$

In Eq. (3), *Q*_*i*_, *K*_*i*_, and *V*_*i*_ denote the mapping matrices of the original input to the *i*-th subspace.

### BiLSTM model and its bidirectional sequence modeling

**Figure 3:**
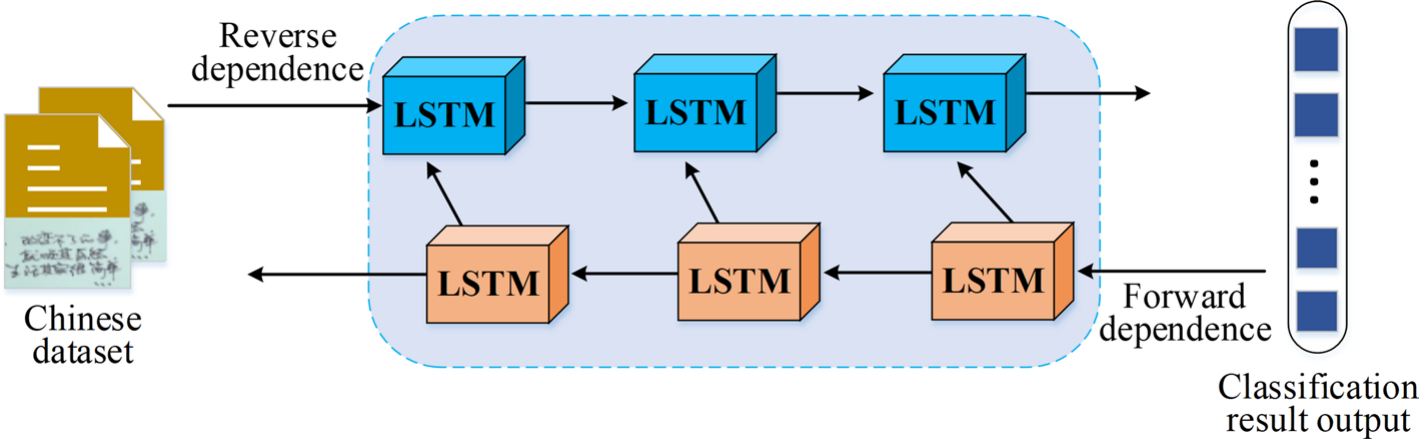
Flow of BiLSTM applied to NLP.

BiLSTM, a variant of the Recurrent Neural Network, operates on the principle of bi-directionality. In the context of a given sentence, its tokens are arranged in a left-to-right order. The distinguishing feature of BiLSTM lies in its utilization of two LSTM networks that function concurrently on both the original sequence (left-to-right) and the reverse sequence (right-to-left). This bidirectional modeling allows the model to consider both the input sequence’s context information and better capture the semantic relationships and mutual influences between word meanings^32,33^. The specific application of BiLSTM in NLP is illustrated in Fig. 3.

When applying the BiLSTM algorithm in NLP, the disambiguation features (*x*_*i*_ and* h*_*t*-1_) are fed into the input of the BiLSTM model. The input data passes through a sigmoid function to obtain the coefficients (*f*_*t*_ and *i*_*t*_). Subsequently, the input is processed through an activation function to obtain the temporary cell variable ($\tilde{c}_{t}$). The computation process is illustrated by Eqs. (4)–(6).

4 $$f_{t} = \delta \left( {\left[ {w_{f} \cdot \left[ {h_{t - 1} ,x_{t} } \right] + b_{f} ,w_{f}{\prime} \cdot \left[ {h_{t - 1}{\prime} ,x_{t} } \right] + b_{f}{\prime} } \right]} \right)$$

5 $$i_{t} = \delta \left( {\left[ {w_{i} \cdot \left[ {h_{t - 1} ,x_{t} } \right] + b_{i} ,w_{i}{\prime} \cdot \left[ {h_{t - 1}{\prime} ,x_{t} } \right] + b_{i}{\prime} } \right]} \right)$$

6 $$\tilde{c}_{t} = \tanh \left( {\left[ {w_{c} \cdot \left[ {h_{t - 1} ,x_{t} } \right] + b_{c} ,w_{c}{\prime} \cdot \left[ {h_{t - 1}{\prime} ,x_{t} } \right] + b_{c}{\prime} } \right]} \right)$$

where *w* stands for the weight matrix, and *b* refers to the bias vector.

Then, the hidden state *h t* at time *t* can be expressed as Eqs. (7)–(9).

7 $$\vec{h}_{t} = \overrightarrow {LSTM} \left( {\vec{w}_{t} ,\vec{h}_{t - 1} ,\vec{b}_{t} ,\vec{c}_{t - 1} } \right)$$

8 $$\mathop{h}\limits^{\leftarrow} _{t} = \overleftarrow {LSTM} \left( {\mathop{w}\limits^{\leftarrow} _{t} ,\mathop{h}\limits^{\leftarrow} _{t - 1} ,\mathop{b}\limits^{\leftarrow} _{t} ,\mathop{c}\limits^{\leftarrow} _{t + 1} } \right)$$

9 $$h_{t} = \left[ {\vec{h}_{t} ,\mathop{h}\limits^{\leftarrow} _{t} } \right]$$

where *W* and* b* represent the relevant weights of the gate unit and the memory cell; *c*_*t*_ and *h*_*t*_ denote the state of the memory cell and the value of the hidden state of the LSTM at *t*, respectively; → and ← represent forward text and reverse text, respectively.

Finally, the Softmax function is used to classify the Chinese word sense data information, as shown in Eq. (10).

10 $$Soft\max_{i} = \frac{{e^{{\left( {W^{\prime} \cdot flatten\left( {M*} \right) + b^{\prime}} \right)}} }}{{\sum\limits_{C} {e^{{\left( {W^{\prime} \cdot flatten\left( {M*} \right) + b^{\prime}} \right)}} } }}$$

In Eq. (10), $W^{\prime}$ refers to the weight matrix of the fully connected layer; $flatten\left( {M*} \right)$ represents stretching the M* matrix into a vector; $b^{\prime}$ signifies the connection bias item of the fully connected layer; *i* indicates the index of the classification category; *C* stands for the total number of categories. The calculation result vector referred to $Soft\max_{i}$ is the probability obtained for the *i*-th word sense category.

### Construction of Chinese word sense recognition model fusing transformer and BiLSTM algorithms

**Figure 4:**
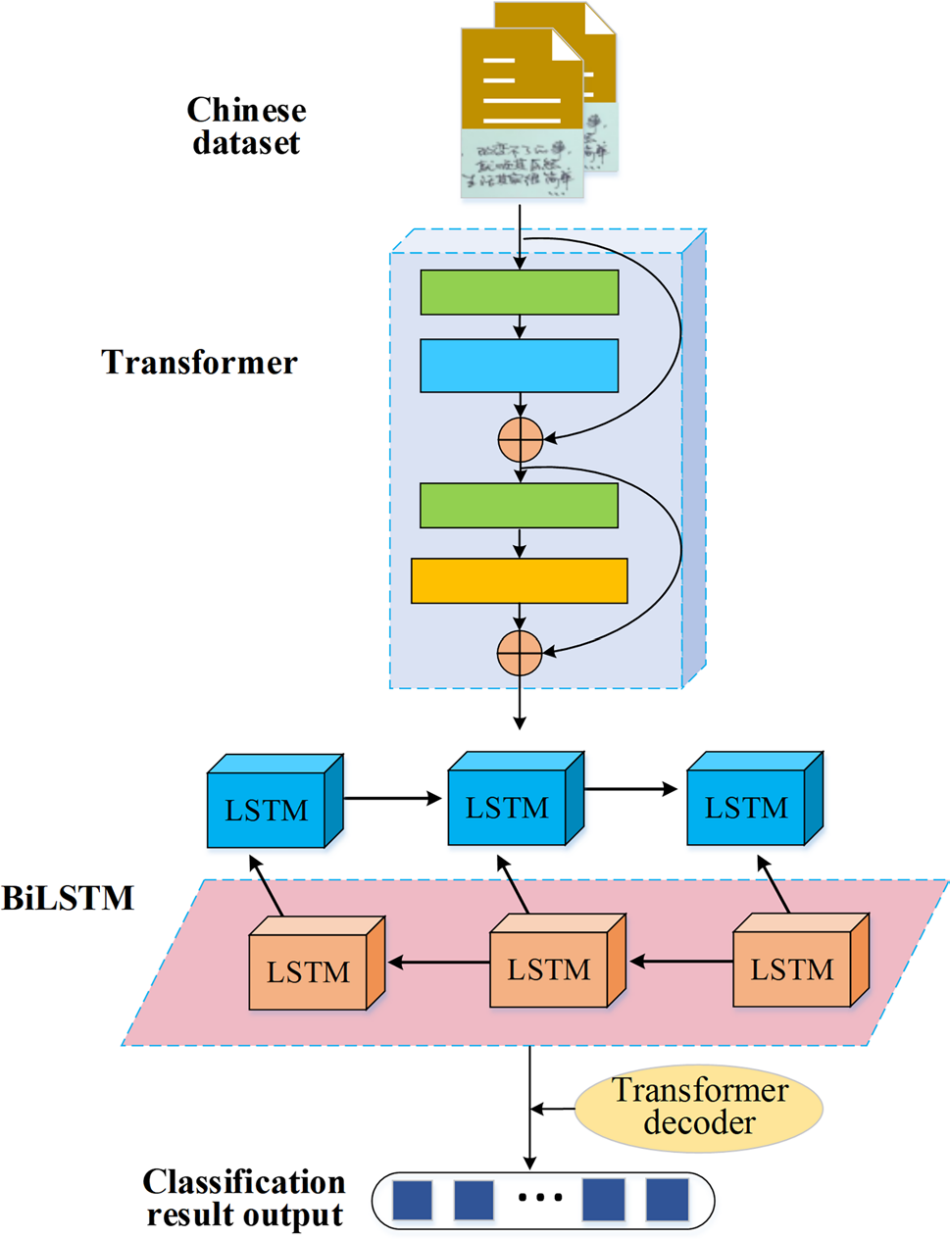
Framework of the Transformer-fused BiLSTM-based Chinese word sense disambiguation model.

The word sense disambiguation task involves a close semantic relationship between the context of ambiguous words and the target word, necessitating the disambiguation model to extract semantic information from the context. This study introduces the Transformer algorithm from deep learning and integrates it with the BiLSTM algorithm to address Chinese word sense disambiguation. By leveraging the self-attention mechanism of the Transformer and the sequence modeling capability of BiLSTM, the model efficiently captures semantic information and context relationships in Chinese sentences, leading to accurate word sense disambiguation. The Transformer-fused BiLSTM-based Chinese word sense disambiguation model is depicted in Fig. 4.

As depicted in Fig. 4, the system model begins with tokenizing and encoding the original Chinese sentence to obtain word vector representations, which serve as inputs to the model. The word vectors then undergo the Transformer encoder layer, comprising multiple attention heads (self-attention heads). These attention heads efficiently capture global semantic information based on word relationships. Subsequently, the BiLSTM layer is applied. Within the BiLSTM layer, bidirectional LSTM networks process the input sentence in both forward and backward directions, capturing contextual information from preceding and succeeding words. Next, the outputs of the Transformer encoder layer and the BiLSTM layer are fused through concatenation and weighted averaging. This fusion process combines the global relationships captured by the Transformer with the bidirectional contextual modeling capability of BiLSTM, resulting in a more comprehensive understanding of the semantic information within the sentence. Finally, the fused features are projected through a fully connected layer and passed through the Softmax function, generating the probability distribution of each word sense label.

Assuming the Transformer encoder consists of N layers, each layer outputs an encoded representation. The output of the *i*-th layer is computed as shown in Eq. (11):

11 $$\left[ {{\text{Transformer}}_{{{\text{encoder}},i}} \left( X \right){\text{ = Norm}}\left( {X{\text{ + Dropout}}\left( {{\text{F}}\left( {{\text{Norm}}\left( X \right)} \right)} \right)} \right)} \right]$$

In Eq. (11), (X) represents the data input to the Transformer encoder, (F) denotes the function of the Transformer encoder layer, which includes self-attention mechanisms and feedforward neural networks, (Norm) represents layer normalization, and (Dropout) is a random dropout operation used to prevent overfitting.

The output of the final layer of the Transformer encoder is computed as shown in Eq. (12):

12 $$\left[ {{\text{Transformer}}\,{\text{final}}\left( X \right){\text{ = Transformer}}\,{\text{encoder}},N\left( X \right)} \right]$$

For the BiLSTM layer, its output depends on the concatenation of the outputs from the forward LSTM and backward LSTM. Assuming the BiLSTM has M layers, the output of the j-th layer is given by Eq. (13):

13 $$\left[ {{\text{BiLSTM}}_{j} \left( X \right){ = }\left[ {\overrightarrow {{{\text{LSTM}}_{j} }} \left( X \right);\overleftarrow {{{\text{LSTM}}_{j} }} \left( X \right)} \right]} \right]$$

In Eq. (13), $(\overrightarrow {{{\text{LSTM}}_{j} }} (X))$ represents the output of the forward LSTM, and $(\overleftarrow {{{\text{LSTM}}_{j} }} (X))$ represents the output of the backward LSTM. ([;]) denotes the concatenation operation.

The output of the final layer of the BiLSTM is expressed in Eq. (14):

14 $$\left[ {{\text{BiLSTM}}_{{{\text{final}}}} \left( X \right) = {\text{BiLSTM}}_{M\left( X \right)} } \right]$$

The outputs of the Transformer encoder and BiLSTM are fused using a simple approach, such as concatenation or weighted averaging. The concatenation method is used as shown in Eq. (15):

15 $$\left[ {{\text{Fusion}}\left( X \right) = \left[ {{\text{Transformer}}\,{\text{final}}\left( X \right);{\text{BiLSTM}}\,{\text{final}}\left( X \right)} \right]} \right]$$

The fused output (Fusion(*X*)) can then be further passed to a fully connected layer for classification or other tasks.

### Simulation experiment evaluation

The implementation was carried out using the TensorFlow framework software to evaluate the performance of the Transformer-fused BiLSTM-based Chinese word sense disambiguation model. The software was executed on a 64-bit Linux operating system and utilized the OpenCV 4.2.0 image processing library and Python version 3.7. The hardware setup comprised an Intel Core i7-8700 K CPU, 512 GB SSD storage, 16 GB RAM, and Nvidia GeForce GTX 1080 Ti GPU. The experimental data for the study was sourced from the PKU-Paraphrase-Bank (https://github.com/pkucoli/PKU-Paraphrase-Bank/), and a total of 20,725 pairs of sentences were collected after applying data cleaning and preprocessing measures to handle noise, redundancy, and duplicate data. This study implemented an enhanced dataset split to evaluate model performance comprehensively and validate research findings. The adjustment included the introduction of a validation set and modifying the dataset split ratio to 70% for the training set, 20% for the validation set, and 10% for the test set. The rationale behind this modification is twofold. First, it facilitates the model training process by using the training set to fit the model, allowing it to learn the features and patterns of the data. Second, it enables the assessment of the model’s performance on unseen data using the test set, thereby detecting potential issues such as overfitting and evaluating the model’s generalization ability. During the model training process, particular attention was given to hyperparameter tuning to ensure optimal performance in various aspects. A grid search method explored and adjusted hyperparameters, including learning rate, batch size, and model structure. The final hyperparameter combination, determined by comparing performance on the validation set, consisted of a learning rate of 0.001 and a batch size of 64, with four layers for the Transformer and two layers for BiLSTM. This meticulous approach to dataset split and hyperparameter tuning enhances the reliability and robustness of the model, ensuring that it is well-adapted to diverse scenarios. The outcomes of hyperparameter tuning are summarized in Table 2:

**Table 2:** Hyperparameter tuning results.

Learning rate	Batch size	Transformer layers	BiLSTM layers	Validation accuracy (%)	Test accuracy (%)	Final model performance
0.0005–0.0015	32–96	4	2	88.50	86.20	Optimal
0.005–0.02	16–64	6	3	85.30	82.70	Moderate
0.05–0.2	64–256	8	4	79.80	76.50	Poor

To assess the performance of the constructed model, a series of comparative experiments were conducted, including comparisons with BiLSTM^34^, Transformer^35^, and recent models proposed by Zheng et al^36^. and Hou et al^37^. The primary evaluation metric utilized in this study was accuracy, which quantifies the proportion of correct predictions made by the model for the word sense disambiguation task. Additionally, to comprehensively evaluate the methods’ performance and stability, other metrics considered in this study were root mean square error (RMSE) and loss value. The accuracy is calculated according to Eq. (16).

16 $$Acc = \frac{TP + TN}{{TP + FP + TN + FN}}$$

In Eq. (16), *TP* represents the number of positive samples predicted to be positive; *FP* denotes the number of negative samples expected to be positive; *FN* refers to the number of positive samples predicted to be negative; *TN* stands for the number of negative samples predicted to be negative.

Equation (17) describes RMSE.

17 $$RMSE = \sqrt {\frac{1}{N}\sum\limits_{t = 1}^{N} {\left| {x_{i} - y_{i} } \right|^{2} } }$$

In Eq. (17), *x*_*i*_ denotes the actual Chinese text data at the *i*-th moment,* y*_*i*_ signifies the Chinese text prediction data output by the model at the *i*-th moment, and* N* refers to the length of the Chinese text data sequence to be evaluated.

## Results and discussions

### Performance comparison results of models under different algorithms

**Figure 5:**
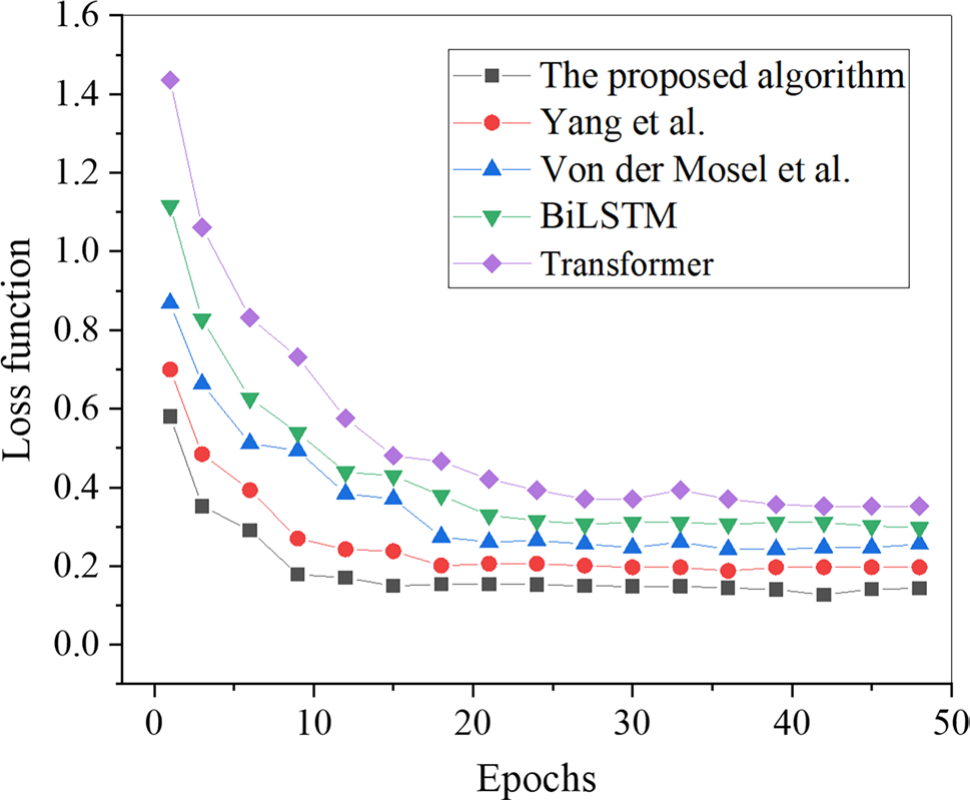
Loss function results.

Figure 5 presents the loss function results of the models under different algorithms.

Figure 5 illustrates the analysis of loss values for various algorithms. Evidently, the proposed Transformer-fused BiLSTM-based model achieves the minimum loss value and reaches a relatively stable state at the 15th iteration cycle, maintaining around 0.14. In contrast, the loss functions of other algorithms all exceed 0.19. As a result, all algorithms converge during the Chinese word sense disambiguation task, and the proposed Transformer-fused BiLSTM-based Chinese word sense disambiguation model demonstrates superior convergence performance with lower loss values. This outcome underscores the supremacy of the Transformer-enhanced BiLSTM model in encoding semantic information and contextual relationships. The Transformer’s self-attention mechanism excels at capturing long-range dependencies, complemented by BiLSTM’s proficiency in sequential modeling. This synergistic integration enables the model to adeptly capture semantic nuances in Chinese sentences, reducing the loss value. The combined strength of these two architectures contributes to the model’s heightened performance in Chinese word sense disambiguation tasks.

**Figure 6:**
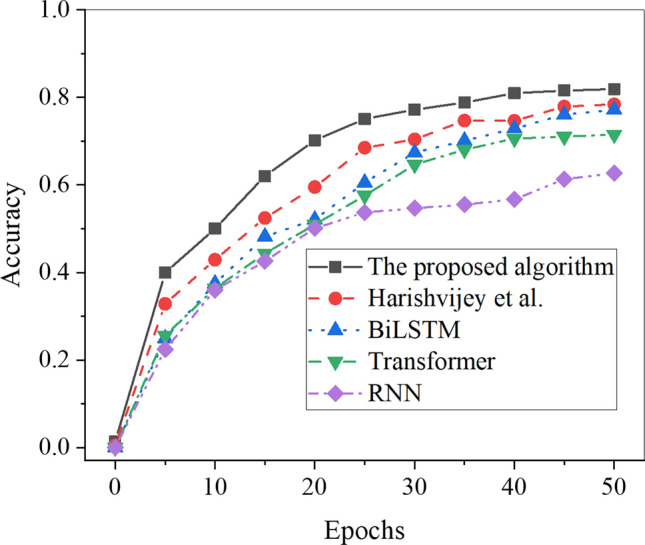
Accuracy results of each algorithm in the training set.

**Figure 7:**
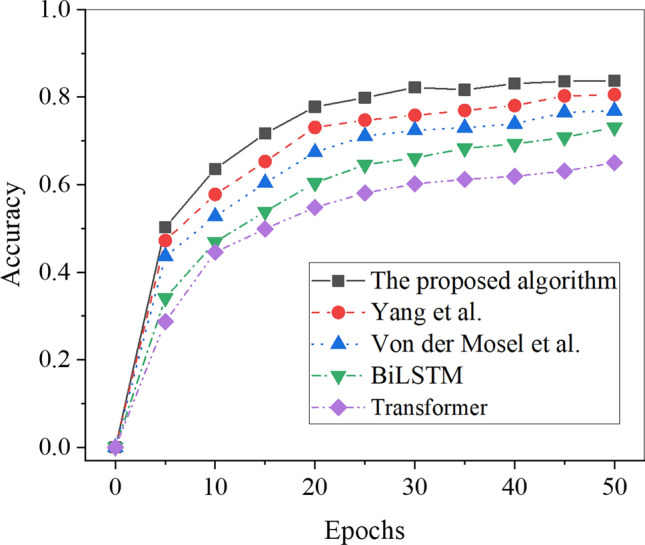
Accuracy results of each algorithm in the test set.

Furthermore, the recognition accuracy of different algorithms on the training and testing datasets is analyzed and depicted in Figs. 6 and 7.

Figure 6 demonstrates that as the number of iterations increases, the accuracy of different algorithms on the training set rapidly improves and stabilizes. Notably, the proposed Transformer-fused BiLSTM-based model achieves a recognition accuracy of 81.91%, which is at least 3.5% higher than the accuracy achieved by other model algorithms. The recognition accuracy of the algorithms for Chinese word sense disambiguation ranks as follows, from highest to lowest: the proposed Transformer-fused BiLSTM-based model, the model algorithm by Hou et al., the model algorithm by Zheng et al., BiLSTM, and Transformer. Consequently, the Transformer-fused BiLSTM-based model exhibits superior accuracy in identifying Chinese word senses in a shorter period.

The analysis of the recognition accuracy of different algorithms on the test set is depicted in Fig. 7. As the number of iterations increases, the accuracy rapidly increases, followed by stabilization. Notably, the proposed Transformer-fused BiLSTM-based model achieves a recognition accuracy of 83.71% for Chinese word sense disambiguation on the test set, which surpasses the accuracy achieved by other model algorithms by at least 3.05%. Furthermore, the recognition accuracy of the algorithms for Chinese word senses, in descending order, is as follows: the proposed Transformer-fused BiLSTM-based model, the model algorithm by Hou et al., the model algorithm by Zheng et al., BiLSTM, and Transformer. Consequently, the Transformer-fused BiLSTM-based model exhibits superior recognition accuracy in Chinese word sense disambiguation. The observed superiority depicted in Figs. 6 and 7 can be attributed to several pivotal factors. Firstly, as the number of iterations increases, the Transformer-enhanced BiLSTM model consistently exhibits heightened accuracy on both the training and testing sets. This performance can be primarily ascribed to the superior model structure, adeptly leveraging the self-attention mechanism of the Transformer and the sequential modeling advantages inherent in BiLSTM. This synergy enables the model to effectively capture semantic nuances and contextual relationships in Chinese sentences, thereby elevating recognition accuracy. Secondly, post-parameter tuning, the model showcases enhanced performance across the training and testing sets. The adjustment of hyperparameters to better align with the intricacies of the Chinese language context contributes to an overall performance boost. Lastly, the Transformer-enhanced BiLSTM model, amalgamating two distinct neural network structures, achieves a harmonious equilibrium between the demands of long-distance dependency and sequential modeling. This delicate balance markedly enhances the overall effectiveness of the algorithm.

**Figure 8:**
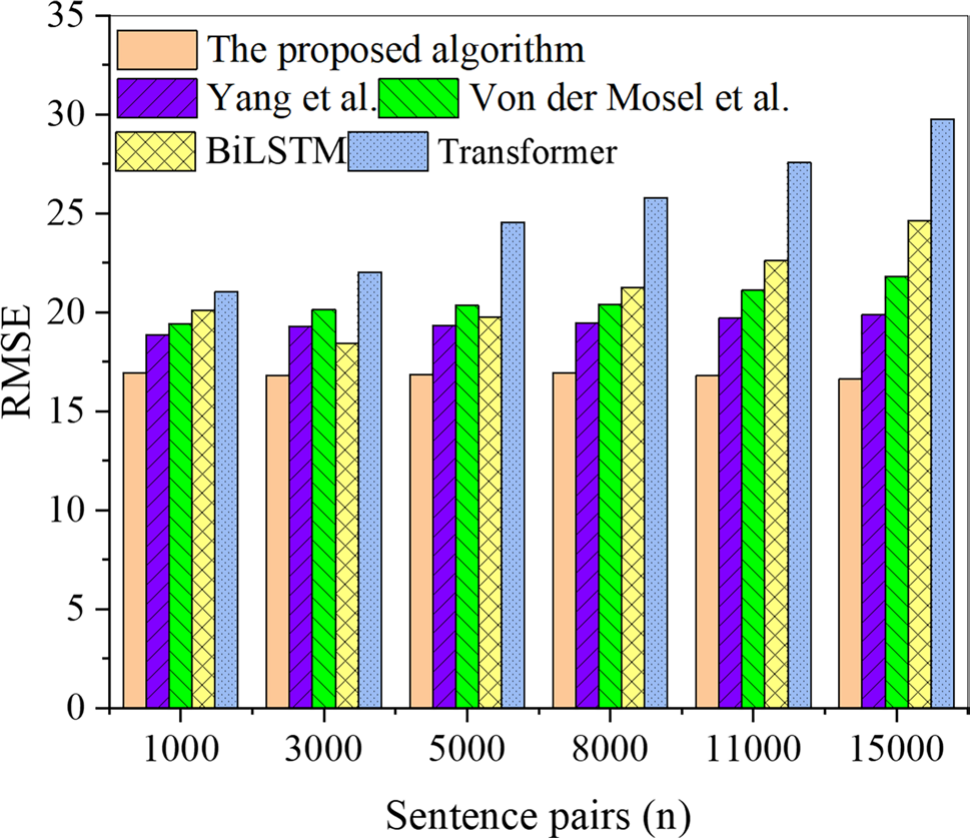
RMSE results of each algorithm in the training set under different sentence pairs.

**Figure 9:**
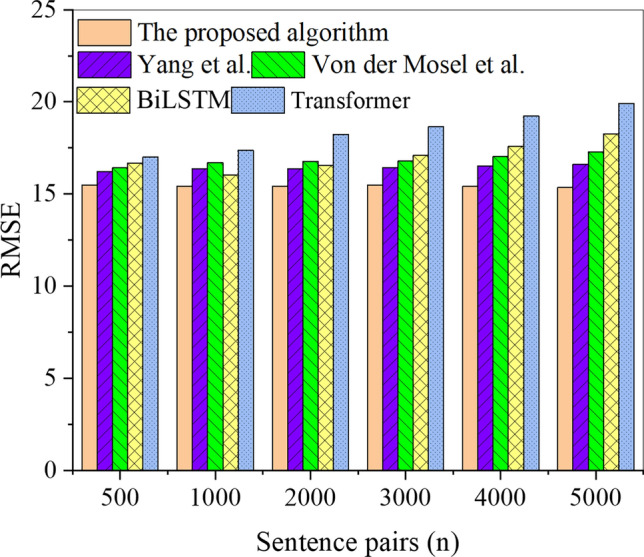
RMSE results of each algorithm in the test set under different sentence pairs.

Additionally, a comparative analysis of the RMSE for different algorithms on both the training and test sets is presented in Figs. 8 and 9, respectively.

In Figs. 8 and 9, the analysis of the RMSE results for different algorithms on the training and test sets indicates a slight upward trend in RMSE with the increase in sentence pairs, but the trend is not significant. In the training set, the proposed Transformer-fused BiLSTM-based model achieves the highest RMSE of 16.95, while the RMSE for other model algorithms is more elevated, exceeding 18.88. Similarly, in the test set, the highest RMSE for the proposed Transformer-fused BiLSTM-based model is 15.47, and the RMSE for other model algorithms is also higher than that of this paper’s model algorithm. The RMSE results of each algorithm, sorted from the smallest to the largest, are as follows: the proposed Transformer-fused BiLSTM-based model, the model algorithm by Hou et al. (2020), the model algorithm by Zheng et al. (2021), BiLSTM, and Transformer. Consequently, the Transformer-fused BiLSTM-based model demonstrates lower classification and recognition errors for Chinese word sense disambiguation. The meticulous analysis of the trend in RMSE, as illustrated in Figs. 8 and 9, unveils several salient factors. Firstly, within the training set, the proposed Transformer-enhanced BiLSTM model showcases a notably low RMSE (16.95), underscoring its supremacy in comprehending Chinese sentence contexts. Its inherent sequential modeling prowess empowers it to adeptly capture contextual nuances, thereby mitigating classification and recognition errors. Comparative to alternative models, its diminished RMSE signifies superior performance on the training set. In the testing set, the proposed Transformer-enhanced BiLSTM model similarly demonstrates a low RMSE (15.47), affirming its superiority over alternative model algorithms. This advantage underscores the model’s capability to sustain a low error level when confronted with previously unseen data, thereby affirming its commendable generalization ability. Secondly, the overall trend indicates a marginal increase in RMSE with a corresponding escalation in the number of sentence pairs; however, this trend is not pronounced. This increase attests to the model’s relative robustness in the face of a growing corpus of contexts and sentence pairs. The model’s flexible contextual modeling imparts resilience, ensuring consistent performance across varying data scales.

### Performance of the transformer-fused BiLSTM Chinese word sense disambiguation model on different standard benchmarks

The performance evaluation of the proposed model on various standard benchmarks is detailed in Table 3. The Transformer-fused BiLSTM Chinese Word Sense Disambiguation model effectively tackles word sense ambiguity in Chinese contexts across diverse benchmarks. The model attains commendable accuracy on the PKU Phrase Bank and Chinese Vocabulary Semantic Benchmark, achieving 83.71% and 84.47%, respectively. This result underscores the model’s capacity for accurate prediction in disambiguating word senses within Chinese sentences. The synergistic integration of Transformer and BiLSTM models empowers the system to adeptly capture semantic intricacies and contextual relationships, thereby amplifying disambiguation effectiveness. On the SENSEVAL-3 Chinese Dataset, the model achieves an accuracy of 76.93%, marginally lower than other benchmarks. Several factors may contribute to this comparatively lower performance. The dataset encompasses texts with unique contexts and language structures, introducing heightened complexity and including contextual information that may pose challenges for the model. Additionally, specific domain differences in the dataset compared to other benchmarks might impact performance. Inadequate coverage of linguistic characteristics of this domain during training could lead to suboptimal performance. Enhancing domain adaptation becomes imperative for optimal performance on the SENSEVAL-3 dataset. The accuracy of dataset annotations plays a pivotal role in model performance. Inaccurate or ambiguous annotations in the SENSEVAL-3 Chinese dataset can detrimentally affect the model, resulting in diminished performance. Rigorous validation and cleaning of annotations are imperative to ensure label accuracy. Furthermore, if the training set lacks coverage of specific contexts or word senses present in the SENSEVAL-3 Chinese dataset, the model’s generalization to these situations might be compromised. In such scenarios, augmenting the diversity and coverage of the training set becomes crucial for enhancing model performance on the SENSEVAL-3 dataset. Moreover, the model’s architecture may exhibit varying performance on distinct types of contexts or tasks. If the model structure inadequately adapts to the characteristics of the SENSEVAL-3 Chinese dataset, its performance could be constrained. Exploring adjustments to the model structure or considering a model better suited to this dataset represents a potential avenue for improvement.

**Table 3:** Performance of the model on different standard benchmarks.

Standard benchmark	Accuracy (%)	Recall (%)	Precision (%)	F1 (%)	RMSE	Loss
PKU phrase bank	83.71	83.33	95.24	88.89	16.95	0.14
Chinese vocabulary semantic Benchmark ^36^	84.47	88.89	92.31	90.57	17.69	0.39
SENSEVAL-3 Chinese dataset ^38^	76.93	78.26	85.71	81.52	19.83	0.68

Nevertheless, the model demonstrates relatively high recall rates across all standard benchmarks, reaching 83.33%, 88.89%, and 78.26% for PKU Phrase Bank, Chinese Vocabulary Semantic Benchmark, and SENSEVAL-3 Chinese Dataset, respectively. These outcomes underscore its efficacy in capturing positive samples within the text. In terms of precision, the model achieves 95.24% and 92.31% on the PKU Phrase Bank and Chinese Vocabulary Semantic Benchmark, respectively, while attaining 85.71% on the SENSEVAL-3 Chinese Dataset. Striking a balance between recall and precision, the F1 values are 88.89% and 90.57% for the PKU Phrase Bank and Chinese Vocabulary Semantic Benchmark, respectively, and 81.52% for the SENSEVAL-3 Chinese Dataset. The model’s performance is also further assessed through RMSE and loss function values across all benchmarks. Collectively, these results highlight the superiority of the Transformer-fused BiLSTM model in Chinese Word Sense Disambiguation tasks, providing robust support for semantic understanding in the Chinese context.

### Discussion

This study designed and implemented a novel model algorithm that combines Transformer and BiLSTM for Chinese word sense disambiguation. The performance of this model algorithm was evaluated by comparing it with other existing algorithms, namely Hou et al., Zheng et al., BiLSTM, and Transformer. Uniform parameter settings were consistently applied throughout the comparison process to ensure a fair comparison. All models were configured with identical parameters, setting the learning rate to 0.001 and batch size to 64, among other specifications. This approach mitigates performance differences that may arise from disparate parameter configurations, thereby ensuring the fairness and validity of the comparison. The results indicate that the Transformer-fused BiLSTM-based model outperformed other algorithms, particularly in terms of loss values. It attained a stable state with a notably low loss value of approximately 0.14 by the 15th iteration cycle. In the 15 iteration cycles, the BiLSTM model exhibited a loss value of 0.30, while the Transformer model showed a loss value of 0.35. This signifies a notable improvement of over 0.05 compared to the other algorithms. These findings highlight that all algorithms can achieve convergence in the Chinese word sense disambiguation task and demonstrate relatively lower loss values, which aligns with the observations made by Özçift et al^39^.

Further analysis of recognition accuracy revealed that the Transformer-fused BiLSTM model achieved outstanding recognition accuracies of 81.91% and 83.71% on the training set and testing set, respectively. In comparison, the BiLSTM model achieved recognition accuracies of 77.17% on the training set and 73.10% on the test set, while the RNN model recorded recognition accuracies of 62.70% on the training set and 65.00% on the test set. Notably, these accuracies surpassed those of other model algorithms, including Hou et al., Yang et al., Zheng et al., Von der Mosel et al., BiLSTM, and Transformer. Furthermore, in terms of RMSE, the Transformer-fused BiLSTM-based model exhibited lower values, recording 16.95 on the training set and 15.47 on the testing set. In comparison, the BiLSTM model showed RMSE values of 24.60 on the training set and 18.26 on the test set, while the Transformer model displayed RMSE values of 29.75 on the training set and 19.91 on the test set. Notably, all other model algorithms registered higher RMSE values. This finding indicates that the Transformer-fused BiLSTM-based model exhibits lower classification and recognition errors in Chinese word sense disambiguation. These observations align with the views presented by Ji et al^40^. and Chen et al^41^.

Therefore, the Transformer-fused BiLSTM-based Chinese word sense disambiguation model demonstrated excellent performance in terms of loss values, accuracy, and RMSE. The results highlight the model’s high accuracy and robustness in Chinese word sense understanding tasks, providing a strong reference for advancing the development of intelligent Chinese word sense disambiguation.

## Conclusion

This study introduces deep learning algorithms to efficiently and accurately identify word senses in the Chinese language. A Chinese word sense disambiguation model is constructed based on the fusion of Transformer and BiLSTM. The experimental analysis of the model’s performance demonstrates that the proposed Transformer-fused BiLSTM-based model outperforms other methods in terms of loss values, accuracy, and RMSE, achieving a recognition accuracy of approximately 83%. This result indicates superior convergence and recognition accuracy, showcasing the model’s significant advantages in Chinese word sense understanding tasks.

However, there are some limitations in this study. This study has several limitations that warrant consideration. Firstly, the primary focus of this research is on Chinese word sense disambiguation, mainly through the integration of Transformer and BiLSTM. The effectiveness of the proposed methodology may not be as pronounced when applied to word sense disambiguation in languages other than Chinese due to variations in language structure, grammar rules, and linguistic contexts. Secondly, the methodology may exhibit suboptimal performance in handling specific scenarios. Additionally, when dealing with word sense disambiguation in specific domains, the methodology might demand more domain-specific knowledge, which may not be easily acquired from general datasets. Therefore, future research should consider utilizing more diversified Chinese word sense datasets that cover different domains and topics to validate the model’s applicability in broader contexts.

### Supplementary Information

Supplementary Information.

## Data Availability

All data generated or analysed during this study are included in this published article [and its supplementary information files].
